# Supplementary material for: Intention to donate blood and its predictors among adults of Gondar city: Using theory of planned behavior
Source: PLoS One. 2020 Mar 2;15(3):e0228929. doi: 10.1371/journal.pone.0228929 (PMC7051045; doi:10.1371/journal.pone.0228929)
Supplement: Attachment — Submitted filename: Response to editor.docx [file pone.0228929.s001.docx]

To: Editor in Chief of PLOS ONE Journal	Date: August, 29, 2019

**Editors’ comment**

We have significant concerns about the grammar, usage, and overall readability of the manuscript. **Authors’ response**

We edited the whole manuscript based on your comments using professional language editors in our university.
