# Supplementary material for: Intention to donate blood and its predictors among adults of Gondar city: Using theory of planned behavior
Source: PLoS One. 2020 Mar 2;15(3):e0228929. doi: 10.1371/journal.pone.0228929 (PMC7051045; doi:10.1371/journal.pone.0228929)
Supplement: Attachment — Submitted filename: Reviews-PlosOne.docx [file pone.0228929.s002.docx]

**Reviews-PONE-D-19-22717
INTENTION TO DONATE BLOOD AND ITS PREDICTORS AMONG ADULTS OF GONDAR CITY: USING THEORY OF PLANNED BEHAVIOR**

**Line 34**- write the very variables instead of independent and dependent

Study population and sampling

**Line 78** -Mention the very “Outcome variable “you have used, and the parameters used for estimating the sample size

**Line 91-119-**It appears to involve Likert scale, like wise would be appropriate to provide the internal consistency coefficient.? The description on how attitude was measured is confusing to follow, revise the explanation to be clearer to the readers. If possible, use aided graphical presentation. How was it decided the INTENTION to be measured by 3 items, ATTITUDE by five items and e.t.c?? Provide more details for the validity. Since there were summed up, kindly report the expected maximum score to be attained to show “Positive” behavior

**Line 122**- the TPB appears for the first time, write a full word followed with abbreviation in bracket.

Line 123- keep in mind that, Regression analysis enables to calculate the strength and direction of the association

**Line- 145-** add a unit for age “years” after stating their mean and sd

**Line 149-150**- If you have the reference of the method you have used, cite it. Otherwise make it clear how were questions validated together with a cut-off point of measuring the knowledge.

**Line 151**-Was the knowledge measured on ordinal scale? (High, Low, poor, e.t.c). Otherwise it contradicts to previous line; good vs poor.

**Line 170-171-** Knowledge level is not featured in the table

**Line 216-219-** It will be important the author to explain how was the model built up? what were the criteria of entering and elimination the variables into the model? Furthermore, would be important to show the crude analysis result of each variables remained in the final multiple regression model. Moreover, it would be more informative to the reader if the number of participants involved in the analysis is shown, with their respective row percentage of each variable

**216-236-** Since the **INTENTION TO DONATE BLOOD** was summation of scores from three items, would you consider centering it to the mean to have appropriate interpretation during regression analysis?

**243**- This is ambiguous sentence, is not clear what is compared here.

**247-254:** I suggest the discussion to focus more on main objective of the study. The descriptive findings should be ignored.

**255-258-** Would be more informative to write the findings rather than the results which are already on result section. This should be reflected to other findings in discussion section.
