# Supplementary material for: Intention to donate blood and its predictors among adults of Gondar city: Using theory of planned behavior
Source: PLoS One. 2020 Mar 2;15(3):e0228929. doi: 10.1371/journal.pone.0228929 (PMC7051045; doi:10.1371/journal.pone.0228929)
Supplement: Attachment — Submitted filename: PONE D 19 22717 review.docx [file pone.0228929.s003.docx]

**Summary:** Authors used theory of planned behavior to understand the intention towards blood donation among Africans. They found attitude, subjective norm, perceived behavior control and past blood donation as significant predictors of blood donation.

**General comments**: English editing is required and proper citations are needed.

**Specific comments:**

**Introduction:**

1. Theory of planned behavior (TPB) should be explained with its 3 parameters attitude (att.), subjective norm (SN) and perceived behavior control (PBC).
2. Studies done on Africans related to blood donation intentions should be cited (For example, Am J Health res 2014; 2:158-63)
3. Rationale should be robust: identify the gaps in previous studies and why there was a need to conduct this study.

**Material & Methods:**

1. The tool with items used for TPB should be provided. Was it an in-house tool? If so, how was it validated?
2. Model of TBP should be explained; were gender, income etc. also entered into the model?
3. How was model fit computed to determine the positive correlation of various parameters with the intention to donate blood?
4. Data collection procedure described intention as one of the variables. This is contrary to the fact that intention is the dependent variable with att., SN and PBC as predictors.
5. For analysis of Likert’s scale, authors used mean and SD. However, if the responses were clustered into two extremes then data may appear neutral. A better way is to provide most frequent responses to each item (or median). A bar chart can be used to illustrate % agree and % disagree.
6. Subjective norm is missing from “measurements”

**Results:**

1. **Table 1** age can be grouped into decades e.g 18-30, 31-40, 41-50 and so on
2. **Table 2:** Methods described using 10 questions for knowledge assessment. However, table 2 provided only 6 questions. A better way of analyzing results for various items is to give % of correct responses.
3. **Past experience of blood donation:** There is a number mismatch; if 15% had experienced donation then 85% had not unlike 94% mentioned in the paper.
4. **Table 3:** this is most confusing. Authors used five-point Likert’s scale so mean should vary from 1-5. The variable used were not explained in methods. Give the reference for estimating indirect subjective norm and also explain its utility.
5. **Magnitude of intention:** What is meant by 44.3% of respondents scored above mean? The intention should be analyzed in context of TPB using regression analysis.
6. **Table 4:** Pearson correlation was used for 7 parameters although table 3 showed 12. Please explain
7. **Table 5:** Please respond to point 2 in material and methods for logical understanding of this table. Please explain negative beta coefficient in the table particularly for those having ‘high knowledge’.

**Discussion:**

1. Need to be re-written in light of above comments
2. Please mention strengths and limitation of the study.
