# Supplementary material for: Intention to donate blood and its predictors among adults of Gondar city: Using theory of planned behavior
Source: PLoS One. 2020 Mar 2;15(3):e0228929. doi: 10.1371/journal.pone.0228929 (PMC7051045; doi:10.1371/journal.pone.0228929)
Supplement: Attachment — Submitted filename: Point by point responses for reviewers TPB.docx [file pone.0228929.s004.docx]

**Point by point responses for reviewers/ editor’s comments**

| **S.No** | **Reviewers/Editor’s comments** | **Authors responses** |
| --- | --- | --- |
| **Reviewed by: Reviewer #1** | | |
| 1 | I am not sure why the authors use regression analysis as this does not allow more complex relationships to be consider and also assumes each variable varies independent of others. Thus Structural equation modelling (which is often used when undertaking research using TPB) would strengthen the analysis. | Dear reviewer thank you for your comments /suggestions.  We conducted linear regression to show association of intention with its predictors and to show relation of each variables with each other including intention we have performed partial correlation. Furthermore, path analysis was conducted for TPB variables to observe relationship of indirect measures with direct measures as well as intention and even to show the relation of indirect variables with each other. |
| 1 | The abstract also mentions that the mean score of giving is lower, but this does not seem to be the focus as the question is how do these other TPB variables influence this intention. The conclusion in the abstract needs to be more than state the results. | Dear reviewer thank you for your comments /suggestions.  We corrected it accordingly.  Please see revised manuscript line 43 |
| 2 | The intro seems to suggest that donation is the only recruitment method and in some African countries people are paid and thus it is not a generous behaviour. | Dear reviewer thank you for your comments /suggestions.  We included it accordingly.  Please see revised manuscript line 63. |
| 3 | I found the introduction and other choppy with multiple single sentence paragraphs. These are all fine, but the authors might craft a story around the issues. | Dear reviewer thank you for your comments /suggestions.  We corrected it accordingly. |
| 4 | For the measures I do think they need to identify the specific scales that were used. These are not referenced and I am not sure who measures were used. This is needed. There is no description of the test of the knowledge measure and how did they make sure their measure was appropriate? For the first question on this it reads as if people could select multiple answers as the sum is greater than the sample number? What did they do with the don’t know respondents are these correct or incorrect? | Dear reviewer thank you for your comments /suggestions.  We have cited the references we used to measure variables. Regarding knowledge variable, we corrected it and if someone responded don’t know answer considered as incorrect, actually we describe the correct answers in each items.  Please see revised manuscript table line 151-154.  Please see revised manuscript Table 3 |
| 5 | It is unclear if they ran a factor analysis to assess the measures or just Cronbach’s alphas? Some constructs don’t mention the alphas. | Dear reviewer thank you for your comments /suggestions.  we used Cronbach’s alpha for internal consistency of items and we corrected by performing if for each items.  Please see revised manuscript line 115-150. |
| 6 | The demographics are fine, but they need to comment if this is reflective of the population. | Dear reviewer thank you for your comments /suggestions. Yes the demographics are reflective of the study population. Researches done in Gondar city also supports these characteristics. |
| 7 | Table 4 does not include knowledge? | Dear reviewer thank you for your comments /suggestions.  We corrected it accordingly.  Please see revised manuscript table 5. |
| 8 | The regression included demographics, which is fine, but thy cold explain this and why it was done. I am not sure why they did not use knowledge as a continuous variable? They also mentioned that they include past donation behaviour and this does not seem to be included and should. | Dear reviewer thank you for your comments /suggestions.  We included demographic variables, past donation experience and knowledge (continuous variable) in regression analysis. We included demographic variables in regression to show “is intention to donate vary among demographic factors?” and the fact we found that they didn’t predict intention.  Please see revised manuscript table 6 |
| 9 | I personally prefer more complex models rather than regression or at least some assessment of interaction effects as it is assumed the variables do not impact on each other. | Dear reviewer thank you for your comments /suggestions.  We conduct path analysis among TPB variables to show interaction of variables each other.  Please see revised manuscript figure 1 |
| 10 | The discussion discuses a range of factors such as fear of needles and I am not sure where this was included in the survey. I do think that there should be a brief discussion of the blood donation process in Ethiopia as it could also be that it I shard to access blood donation locations, which could then explain these results. I just don’t know if it is an issue in the discussion as it is presented. | Dear reviewer thank you for your comments /suggestions.  The factors like fear of needle were assed in indirect measures of perceived behavioral control. We found about five controlling factors during elicitation study conducted prior to the actual survey. and control beliefs were one of the indirect measures of TPB. |
| 11 | The statement “Most individual’s intention to donate blood in the next three months were below the mean intention.” Needs to be rewritten as I think they mean it was below the scale midpoint. Can most people be below average? | Dear reviewer thank you for your comments /suggestions.  We corrected it accordingly.  Please see revised manuscript line 318. |
| 12 **Reviewed by: Reviewer #2** | | |
| 1 | **Line 34**- write the very variables instead of independent and dependent Study population and sampling | Dear reviewer thank you for your comments /suggestions.  We corrected it accordingly.  Please see revised manuscript line 32-34 |
| 2 | **Line 78** -Mention the very “Outcome variable “you have used, and the parameters used for estimating the sample size | Dear reviewer thank you for your comments /suggestions.  We corrected accordingly.  Please see revised manuscript line 99 |
| 3 | **Line 91-119-**It appears to involve Likert scale, likewise would be appropriate to provide the internal consistency coefficient.? The description on how attitude was measured is confusing to follow, revise the explanation to be clearer to the readers. If possible, use aided graphical presentation. How was it decided the INTENTION to be measured by 3 items, ATTITUDE by five items and e.t.c?? Provide more details for the validity. Since there were summed up, kindly report the expected maximum score to be attained to show “Positive” behavior | Dear reviewer thank you for your comments /suggestions.  We incorporated internal consistency for each items in the new manuscript, we presented in table the summary of how the TPB items measured please see table1. We decided number of items for each variables based on TPB manual (Francis *et al.*, 2004).  We included the expected minimum and maximum score in the revised manuscript please see revised manuscript line 115-150. |
| 4 | **Line 122**- the TPB appears for the first time, write a full word followed with abbreviation in bracket. | Dear reviewer thank you for your comments /suggestions.  We corrected it accordingly.  Please see revised manuscript line 160. |
| 5 | Line 123- keep in mind that, Regression analysis enables to calculate the strength and direction of the association | Dear reviewer thank you for your comments /suggestions.  We corrected it accordingly.  Please see the revised manuscript line 161. |
| 6 | **Line- 145-** add a unit for age “years” after stating their mean and sd | Dear reviewer thank you for your comments /suggestions.  We corrected it accordingly.  Please see the revised manuscript line 184. |
| 7 | **Line 149-150**- If you have the reference of the method you have used, cite it. Otherwise make it clear how questions were validated together with a cut-off point of measuring the knowledge. | Dear reviewer thank you for your comments /suggestions.  We corrected it accordingly.  We corrected it accordingly.  Please see the revised manuscript line 152. |
| 8 | **Line 151**-Was the knowledge measured on ordinal scale? (High, Low, poor, e.t.c). Otherwise it contradicts to previous line; good vs poor. | Dear reviewer thank you for your suggestions.  Knowledge measured as continuous variable.  Please see the revised manuscript line 154. |
| 9 | **Line 170-171-** Knowledge level is not featured in the table | Dear reviewer thank you for your comment/suggestions.  We treated knowledge as continuous variable.  please see the revised manuscript line 191-192 |
| 10 | **Line 216-219-** It will be important the author to explain how was the model built up? What were the criteria of entering and elimination the variables into the model? Furthermore, would be important to show the crude analysis result of each variables remained in the final multiple regression model. Moreover, it would be more informative to the reader if the number of participants involved in the analysis is shown, with their respective row percentage of each variable | Dear reviewer thank you for your comment/suggestions.  We corrected it accordingly.  Please see the revised manuscript line 232 244. |
| 11 | **243**- This is ambiguous sentence, is not clear what is compared here. | Dear reviewer thank you for your comment/suggestions.  We corrected it accordingly. |
| 12 | **247-254:** I suggest the discussion to focus more on main objective of the study. The descriptive findings should be ignored. | Dear reviewer thank you for your comment/suggestions.  We removed the descriptive findings like prevalence of past experience of blood donation. please see the revised manuscript. |
| 13 | **255-258-** Would be more informative to write the findings rather than the results which are already on result section. This should be reflected to other findings in discussion section. | Dear reviewer thank you for your comment/suggestions.  We corrected it accordingly.  please see the revised manuscript line 294-305. |
| **Reviewed by: Reviewer #3** | | |
| **introduction** | | |
| 1 | Theory of planned behavior (TPB) should be explained with its 3 parameters attitude (att.), subjective norm (SN) and perceived behavior control (PBC). |  |
|  |  | Dear reviewer thank you for your comment/suggestions.  We corrected it accordingly.  Please see the revised manuscript line 65-75. |
| 3 | Studies done on Africans related to blood donation intentions should be cited (For example, Am J Health res 2014; 2:158-63) | Dear reviewer thank you for your comment/suggestions.  We cited a study conducted in Ethiopia in 2014.  Please see the revised manuscript lin72. |
| 4 | Rationale should be robust: identify the gaps in previous studies and why there was a need to conduct this study. | Dear reviewer thank you for your comment/suggestions.  We corrected it accordingly.  Please see the revised manuscript line 77. |
| **Methods** | | |
| 1 | The tool with items used for TPB should be provided. Was it an in-house tool? If so, how was it validated? | Dear reviewer thank you for your comment/suggestions.  We utilized (I. Ajzen, 2006; Masser *et al.*, 2009; Polonsky, Ferdous and Mcquilten, 2013) and for indirect measures we conduct elicitation study two weeks prior to the survey and those salient beliefs repeated more than 70% were incorporated in to main questionnaire. |
| 2 | Model of TBP should be explained; were gender, income etc. also entered into the model? | Dear reviewer thank you for your comment/suggestions.  Demographic variables entered to the model in the third step. According to TPB model they are stated as external variables. |
| 3 | How was model fit computed to determine the positive correlation of various parameters with the intention to donate blood? | Dear reviewer thank you for your comment/suggestions.  Model fitness were computed for TPB constructs alone, TPB constructs with past experience and over all variables. Please see the R^2^of the model on the revised manuscript line 242, 243 and 256. |
|  | Data collection procedure described intention as one of the variables. This is contrary to the fact that intention is the dependent variable with att., SN and PBC as predictors. | Dear reviewer thank you for your comment/suggestions.  We described all the variables that are used in the study and we classified them independent and dependent variables. We mentioned intention as dependent variable. Please see the revised manuscript line 110-113. |
| 4 | For analysis of Likert’s scale, authors used mean and SD. However, if the responses were clustered into two extremes then data may appear neutral. A better way is to provide most frequent responses to each item (or median). A bar chart can be used to illustrate % agree and % disagree. | Dear reviewer thank you for your comment/suggestions.  We have three direct and three indirect measures of TPB each variables contains four to five items. To show the proportion of for each item using bar graph, it needs more than 28 bar graphs which vague. So we prefer using mean and SD of each variable after summing up of all the items. |
| 5 | Subjective norm is missing from “measurements” | Dear reviewer thank you for your comment/suggestions.  We corrected it accordingly.  Please see the revised manuscript line 133. |
| **Result** | | |
| 1 | **Table 1** age can be grouped into decades e.g 18-30, 31-40, 41-50 and so on | Dear reviewer thank you for your comment/suggestions.  We corrected it accordingly.  Please the revised manuscript line 196. |
| 2 | **Table 2:** Methods described using 10 questions for knowledge assessment. However, table 2 provided only 6 questions. A better way of analyzing results for various items is to give % of correct responses. | Dear reviewer thank you for your comment/suggestions.  We corrected it accordingly.  Please the revised manuscript line 199 |
| 3 | **Past experience of blood donation:** There is a number mismatch; if 15% had experienced donation then 85% had not unlike 94% mentioned in the paper. | Dear reviewer thank you for your comment/suggestions.  Yes, the proportion of past experience is 15% and 85% who had no experience. We didn’t mentioned 94% had experience in the manuscript.  Please see the revised manuscript line 203-208. |
| 4 | **Table 3:** this is most confusing. Authors used five-point Likert’s scale so mean should vary from 1-5. The variable used were not explained in methods. Give the reference for estimating indirect subjective norm and also explain its utility. | Dear reviewer thank you for your comment/suggestions.  We corrected it.  Please the revised manuscript table 1. |
| 5 | **Magnitude of intention:** What is meant by 44.3% of respondents scored above mean? The intention should be analyzed in context of TPB using regression analysis. | Dear reviewer thank you for your comment/suggestions.  We corrected it accordingly.  Please see the revised manuscript line 217. |
| 6 | **Table 4:** Pearson correlation was used for 7 parameters although table 3 showed 12. Please explain | Dear reviewer thank you for your comment/suggestions.  Please the revised manuscript table 4& 5. |
| 7 | **Table 5:** Please respond to point 2 in material and methods for logical understanding of this table. Please explain negative beta coefficient in the table particularly for those having ‘high knowledge’. | Dear reviewer thank you for your comment/suggestions.  We treated knowledge as continuous variable in the revised manuscript and it had no significant relation with intention. If had had significant association and having negative beta coefficient, that means as knowledge score increase the intention of individual to donate blood decreased. However, it had no association we can’t say in such way. |
| **Discussion** | | |
| 1 | Need to be re-written in light of above comments | Dear reviewer thank you for your comment/suggestions.  We corrected it accordingly. |
| 2 | Please mention strengths and limitation of the study. | Dear reviewer thank you for your comment/suggestions.  We describe the strength and limitation of the study.  Please see the revised manuscript line 312. |
