# Supplementary material for: Intention to donate blood and its predictors among adults of Gondar city: Using theory of planned behavior
Source: PLoS One. 2020 Mar 2;15(3):e0228929. doi: 10.1371/journal.pone.0228929 (PMC7051045; doi:10.1371/journal.pone.0228929)
Supplement: Attachment — Submitted filename: Reviews-PlosOne (1).docx [file pone.0228929.s005.docx]

**INTENTION TO DONATE BLOOD AND ITS PREDICTORS AMONG ADULTS OF GONDAR CITY: USING THEORY OF PLANNED BEHAVIOR**

**Line 81;** What does “generous individuals” mean? Use appropriate terminology for blood donations such as Voluntary, Paid, Replacement donors etc.

**Line 82;** Rephrase this sentence to appropriate using blood donation terminology “trend of people going and providing blood in blood banks” voluntary, replacement donation, repeated donation, regular donation, etc.

**Line 180**- the word knowledge should be added on “Respondents were asked ten questions that assess blood donation”, to have a meaningful statement

**Line-194**, The statement is not clear, rephrase it keeping in mind that at this paragraph you are reporting results of knowledge assessment, and not awareness.

**Line 198&201:** Table 2, keeping one decimal place will suffice.

**Line 201-**Table three is not clear, simplify the

**Line 206**-trice should be thrice

**Line 255-232:** What were your cut-off point of Strong and Weak correlation? provide with reference

**Line 258-284;** Revise your interpretation of linear regression, keeping in mind is that for every score of independent variables will expect X point increase of intention score to donate blood. The intention here were measured through scoring

**Line 300**: What is the implication? remove the figures

**Line 328**- You need to explain the implication of those strength and limitation in this study.

**Line 385**- Your study was not on proving if the theory can be used to determine blood donation intention. Focus on the objective of the study.
