# Supplementary material for: Intention to donate blood and its predictors among adults of Gondar city: Using theory of planned behavior
Source: PLoS One. 2020 Mar 2;15(3):e0228929. doi: 10.1371/journal.pone.0228929 (PMC7051045; doi:10.1371/journal.pone.0228929)
Supplement: Attachment — Submitted filename: Point by point response - Copy.docx [file pone.0228929.s006.docx]

**Point by point responses for reviewers/ editor’s comments**

| **S.No** | **Reviewers/Editor’s comments** | **Authors responses** |
| --- | --- | --- |
|  | **Editor’s comments** | |
|  | General comment | Dear Editors thank you for your comments /suggestions. We tried to modify and incorporate all the comments /suggestions raised by the reviewers. Please see all  Components/parts of the revised manuscript. |
|  | I would ask the authors to de-capitalize the title of the manuscript | Dear Editors thank you for your comments /suggestions; We have revised based on your comments. |
|  | **Reviewer#1** | |
| 1 | The amount of unit of blood needed by blood banks and actual collected unit of blood is not compatible. Should be that there is a deficit between the amount of blood needed and collected. | Dear reviewer thanks you for your comments /suggestions. We have revised based on your comments. |
| 2 | In developing countries like Ethiopia the most source of blood is obtained from generous individuals, and most of it is collected through campaign because there is no trend of people going and providing blood in blood banks.” I am not sure what this is saying? | Dear reviewer thanks you for your comments /suggestions. We have revised based on your comments.  Please see the revised manuscript line 80. |
| 3 | Therefore this study assessed the factors within the theory of planned behaviour which determine influence individuals to donate blood; including like their attitude, influence of other individuals (subjective norm), presence of facilitating and hindering factors, individuals level of perceived behavioural control and their previous experience of donation to future. | Dear reviewer thanks you for your comments /suggestions. We have revised based on your comments.  We tried to rephrase it.    Please see the revised manuscript on line 81-83 |
| 4 | The elicitation study was conducted, to explore relevant salient behavioral beliefs, normative belief and perceived behavioral control. | Dear reviewer thank you for your comments /suggestions.  We removed these statement from manuscript because we thought it has little importance there. |
| 5 | five and twenty five respectively. (Other times they us 5 to 25) | Dear reviewer thank you for your comments /suggestions.  We tried to revise it and hope that they are consistent right now.  Please see the revised manuscript on line 110-152. |
| 6 | Knowledge: knowledge towards blood donation was assessed using ten questions which were previously used items in similar research which had similar demographic characteristics. | Dear reviewer thank you for your comments /suggestions.  We corrected it accordingly. |
| 7 | The test for outlier was assessed using Cook's and there was no outlier. (Is this a method or a measure and should be clearer also do they need a reference?) | Dear reviewer thank you for your comments /suggestions.  Linear regression has many assumptions such as linearity, normality, homoscesadisticity and absence of outlier. Therefore, it is one of the assumptions of linear regression. It is checked before fitting the model like other assumptions. We have cited reference for it.  Please the revised manuscript on line 170. |
| 8 | The authors need to indicate what do not know means for the knowledge scale, in the manuscript, not just in the response document (this is also not discussed in the knowledge section and there may be no don’t know category). Knowledge is also not described in Table 1. I am not sure you need to explain response for individual questions, as this appears in Table | Dear reviewer thank you for your comments /suggestions.  In the reviewed manuscript We tried to explain the “don’t know” response and the variable knowledge is included in table 1.  Please see the revised manuscript line 148-155. |
| 9 | Correlation does not look at the relationship (i.e. inferring causality). Thus this might need to be rephrased. | Dear reviewer thank you for your comments /suggestions.  Yes, correlation doesn’t show causality because all relations might not be causality relationship and we didn’t mentioned that there is causality type of relationship.  Dear reviewer we corrected it and make it “correlation”.  Please see the revised manuscript line 222. |
| 10 | In the paper it needs to explain that the sample is representative of the population. I do think this is odd given the composition (i.e. the population is not 66% female and a very high orthodox Christian sample). In the mention of the religion, it should read Christian orthodox means as there are many religions that refer to orthodox? | Dear reviewer thank you for your comments /suggestions.  We used simple random sampling technique to select participants if there were more than one eligible participant in one house. So there is a chance of many females being finally because it is proportional allocation of samples based on sex. Regarding religion, in the study area many of the community members are orthodox Christian follower. Other studies also support this composition. For example, “Mulugeta M. etal,….” |
|  | I want to thank the authors for the additions of the models, but I am not sure how these relate to multiple regression in Table 6? If this is a SEM model, then it needs to be better explained and discussed. If they don’t do this then they should cut the diagram. | Dear Reviewer I thank you for your detailed observation and constructive comments and suggestions.  We tried to re-correct in this revised manuscript. |
| **Reviewed by: Reviewer #2** | | |
|  |  |  |
| Line 81 | What does “generous individuals” mean? Use appropriate terminology for blood donations such as Voluntary, Paid, Replacement donors etc. | Dear reviewer thank you for your comments /suggestions. We have revised based on your comments.  We have corrected it accordingly.  Please see the revised manuscript line 80. |
| Line 82 | Rephrase this sentence to appropriate using blood donation terminology “trend of people going and providing blood in blood banks” voluntary, replacement donation, repeated donation, regular donation, etc. | Dear reviewer thank you for your comments /suggestions. We have rewrite by concentrating on the inclusive terms.  Please on line 78 |
| Line 180 | the word knowledge should be added on “Respondents were asked ten questions that assess blood donation”, to have a meaningful statement | Dear reviewer thank you for your comments /suggestions.  We have added it. |
| Line 194 | The statement is not clear, rephrase it keeping in mind that at this paragraph you are reporting results of knowledge assessment, and not awareness. | Dear reviewer thank you for your comments /suggestions.  We tried to re write it.  Please see line 191 |
| **Line 198&201:** | Table 2, keeping one decimal place will suffice. | Dear reviewer thank you for your comments /suggestions.  We have corrected it accordingly.  Please see 195 |
| **Line 201** | Table three is not clear, simplify the | Dear reviewer thank you for your comments /suggestions.  We have tried to make it simple and could easily be understood.  Please see table 3. |
| **Line 206** | trice should be thrice | Dear reviewer thank you for your comments /suggestions.  We have corrected it accordingly.  Please see line 203. |
| **Line 255 -232** | What were your cut-off point of Strong and Weak correlation? provide with reference | Dear reviewer thank you for your comments /suggestions.  We cite reference for the strength of association.  Please see line 227. |
| **Line 258-284** | Revise your interpretation of linear regression, keeping in mind is that for every score of independent variables will expect X point increase of intention score to donate blood. The intention here were measured through scoring | Dear reviewer thank you for your comments /suggestions.  We have corrected it accordingly |
| **Line 300** | What is the implication? remove the figures | Dear reviewer thank you for your comments /suggestions.  We have removed the figure.  Please see line 274 |
| **Line 328** | You need to explain the implication of those strength and limitation in this study. | Dear reviewer thank you for your comments /suggestions.  We removed strength and limitation part because we found that it has little importance to the paper. |
| **Line 385** | Your study was not on proving if the theory can be used to determine blood donation intention. Focus on the objective of the study. | Dear reviewer thank you for your comments /suggestions.  We have corrected it accordingly. |
